# Supplementary material for: Characterizing cell-type spatial relationships across length scales in spatially resolved omics data
Source: Nat Commun. 2025 Jan 3;16:350. doi: 10.1038/s41467-024-55700-1 (PMC11699133; doi:10.1038/s41467-024-55700-1)
Supplement: Supplementary file 3 — Supplementary Software 1 [file 41467_2024_55700_MOESM3_ESM.zip › CRAWDAD/plots/supplementary/S7_cluster_exp_PKHL_clusters.pdf]

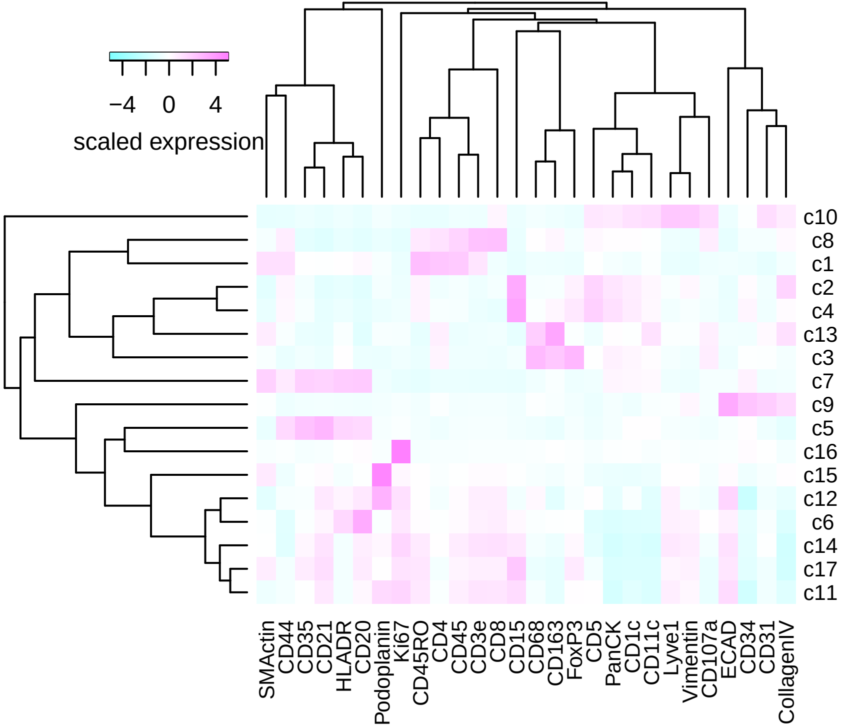
−4 0 4
scaled expression
c10
c8
c1
c2
c4
c13
c3
c7
c9
c5
c16
c15
c12
c6
c14
c17
c11
SMActin
CD44
CD35
CD21
HLADR
CD20
Podoplanin
Ki67
CD45RO
CD4
CD45
CD3e
CD8
CD15
CD68
CD163
FoxP3
CD5
PanCK
CD1c
CD11c
Lyve1
Vimentin
CD107a
ECAD
CD34
CD31
CollagenIV
